# Simultaneous Percutaneous Spinal Cord Stimulation Lead Placement During Spine Surgery

**DOI:** 10.3390/neurosci7050099

**Published:** 2026-09-02

**Authors:** Hiroshi Inagaki

**Affiliations:** Department of Neurosurgery, Spine Center, Seirei Memorial Hospital, Hitachi 319-1235, Ibaraki, Japan; k.ing.16.neurosurgery@gmail.com

**Keywords:** spinal cord stimulation, spine surgery, perioperative neuromodulation, percutaneous lead placement, reversible neuromodulation, retrospective case series

## Abstract

Spinal cord stimulation (SCS) is an established neuromodulatory therapy for refractory chronic pain; however, the timing of SCS initiation has received relatively little attention. In some patients requiring spine surgery, the indication for SCS is already present at the time of surgery. Percutaneous leads combine reversibility with low invasiveness, allowing the timing of SCS introduction to be matched to clinical need. We describe a technical approach for simultaneous percutaneous SCS lead placement during spine surgery and present a retrospective case series of five illustrative cases. This report discusses the significance of this approach from the perspective of SCS timing, invasiveness, and reversibility. In two patients, the therapeutic objective was achieved during the acute postoperative period, and the leads were electively removed without recurrence. In three patients, continued stimulation benefit was observed, and an implantable pulse generator was placed for permanent use. No procedure-related adverse events occurred. Simultaneous percutaneous SCS lead placement during spine surgery may allow neuromodulation to be delivered at the time it is needed, for the duration it is required, and at the relevant spinal level.

## 1. Introduction

Spinal cord stimulation (SCS) is an established treatment for chronic refractory neuropathic pain [1,2]. However, the timing of SCS initiation has received comparatively little attention [2]. In some patients requiring spine surgery, the indication for SCS is already present at the time of the procedure. In cervical ossification of the posterior longitudinal ligament (OPLL), severe preoperative pain may persist postoperatively [3,4,5]. In multilevel degenerative disease, the extent of the surgical wound and degree of neural involvement can make acute postoperative pain a barrier to early mobilization and rehabilitation [6]. In Parkinson’s disease (PD) with concomitant spinal disease, complication and reoperation rates after decompression are higher, making surgical decision-making difficult [7].

In such patients, the timing of SCS relative to spine surgery becomes a relevant clinical question. Existing reports on perioperative SCS have primarily focused on the management of patients who already carry an SCS or intrathecal pump device and are undergoing an unrelated surgical procedure, addressing decisions regarding device retention or removal [8]. Reports addressing intraoperative optimization of SCS lead placement as a standalone procedure also exist [9]. To our knowledge, reports specifically describing the deliberate, simultaneous combination of decompressive spine surgery with percutaneous SCS lead placement under a single anesthetic remain scarce. Percutaneous SCS leads are reversible and minimally invasive, allowing the timing of introduction to be matched to clinical need. We report five patients in whom percutaneous SCS leads were placed simultaneously with spine surgery, and we discuss the significance of this approach from the perspective of SCS timing. This report describes the technical rationale, procedural details, and initial clinical experience. The five cases are presented to illustrate the technique and its potential applications across diverse clinical indications.

## 2. Materials and Methods

### 2.1. Patient Selection and Indication for SCS

Eligible patients had cervical OPLL, multilevel cervical/lumbar degenerative disease, or Parkinson’s disease (PD) with concomitant spinal disease, and met the indication for both decompressive spine surgery and SCS. The indication for SCS was determined in accordance with existing consensus guidelines on patient selection and trial stimulation [2], requiring a pain duration of at least 3 months and neuropathic pain refractory to appropriate pharmacological treatment, having exhausted the conservative treatment options considered clinically appropriate and potentially effective for each patient at the time. All patients presented with a neuropathic pain character. Prior to SCS, all patients had received conservative treatment with medication alone; interventional treatments such as nerve blocks were not performed. Disease duration for each patient is summarized in Table 1 (range: 3–60 months).

### 2.2. Surgical Technique

In all cases, spinal decompression was performed under general anesthesia. Following completion of decompression, percutaneous SCS leads were inserted during the same anesthetic. With the operative field already exposed, the epidural space at the entry site could be directly visualized, facilitating safe introduction of the lead into the epidural space. Lead position was selected so that the spinal region corresponding to the dermatome of the patient’s most severe preoperative pain was maximally covered. No implantable pulse generator (IPG) was placed at this stage; leads were connected to an external stimulator, and subsequent IPG implantation was considered based on the clinical course of each patient. Fluoroscopy was used in all cases to confirm the final position of the distal contacts, particularly when leads were advanced over long spinal segments, and lead position was additionally confirmed postoperatively by CT in all cases. Intraoperative neurophysiological monitoring (e.g., somatosensory evoked potentials) for lead position confirmation was not performed in this series. Lead and stimulator manufacturers are listed in Table 1 (Patient 1: Medtronic; Patients 2–5: Boston Scientific). Sixteen-contact leads were selected to allow flexible programming across a wider spinal range, and stimulation parameters (pulse width, frequency) were individually adjusted for each patient and are detailed in Table 1.

Prophylactic antibiotics were administered intravenously and orally in all cases. The percutaneous lead exit site was managed with an occlusive film dressing, and the wound was visually inspected daily throughout the period of externalization, until either lead removal or permanent implantation. Blood tests were obtained as clinically indicated to monitor inflammatory markers. This study was approved by the institutional ethics committee (approval no. 23-07, approved 18 December 2023), and informed consent was obtained from all patients.

### 2.3. Representative Case (Patient 3)

A 56-year-old man presented with cervical OPLL causing multilevel spinal cord compression. He had been on sick leave for approximately three months and was under psychiatric care for pain-related depression. Preoperative resting neck pain was NRS 10 and the modified Japanese Orthopaedic Association (mJOA) score was 12. Given this patient’s prolonged severe chronic pain, pain-related depression, and work absence, there was substantial concern that postoperative pain would impede early mobilization, rehabilitation, and return to work [3,4,5]. SCS was therefore initiated concurrently with the spinal procedure.

**Figure 1:**
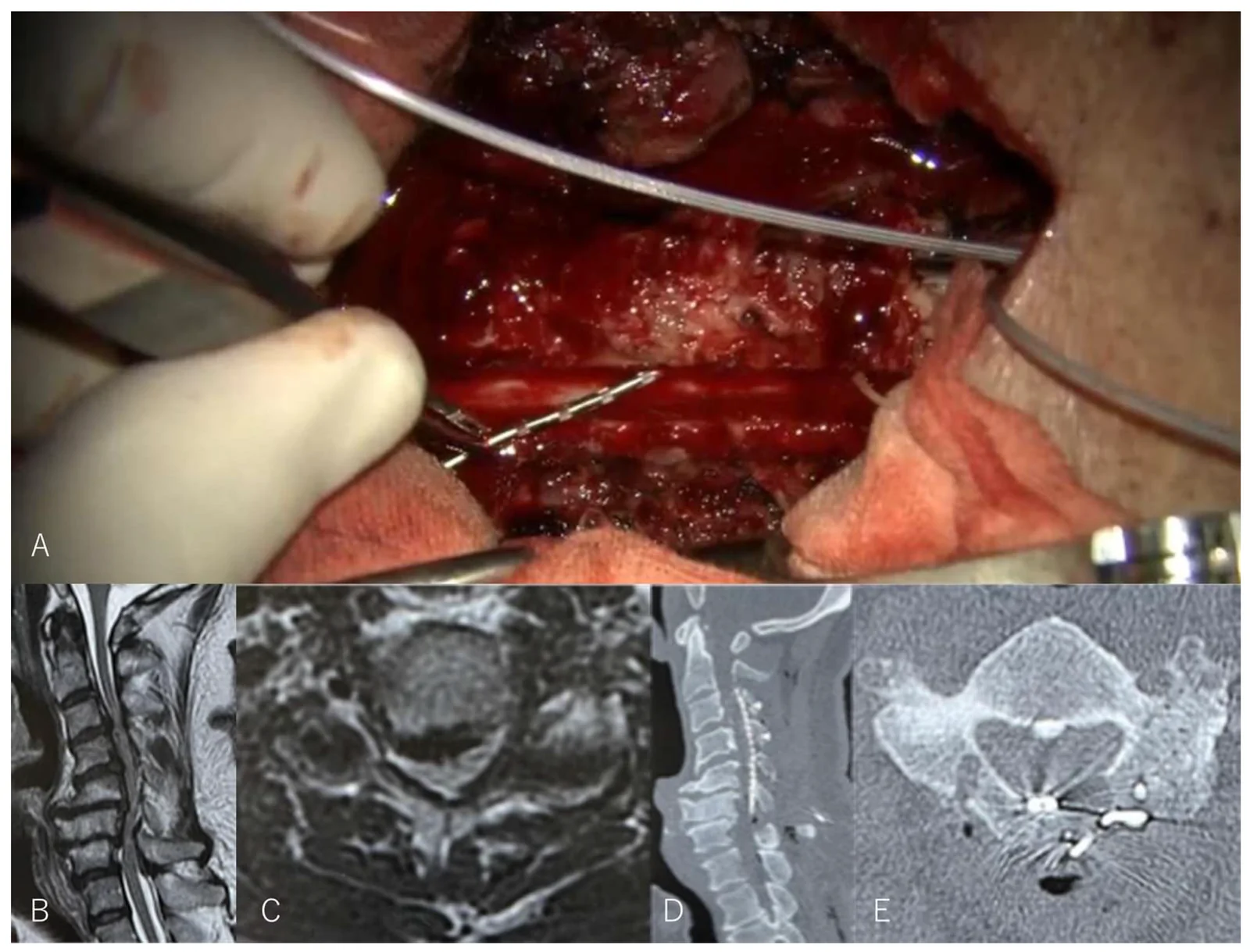
Intraoperative and radiographic findings in the representative case (Patient 3). (**A**) Intraoperative photograph following C2-7 open-door laminoplasty, with the head to the left and the caudal end to the right. After spinal cord decompression, two SCS leads were advanced and positioned in the epidural space by sliding them in a cephalad direction. (**B**) Preoperative T2-weighted MRI (sagittal view) and (**C**) preoperative T2-weighted MRI (axial view) demonstrating multilevel spinal cord compression due to cervical OPLL. (**D**) Postoperative CT (sagittal view) and (**E**) postoperative CT (axial view) confirming bilateral SCS lead placement at the intended levels.

**Figure 2:**
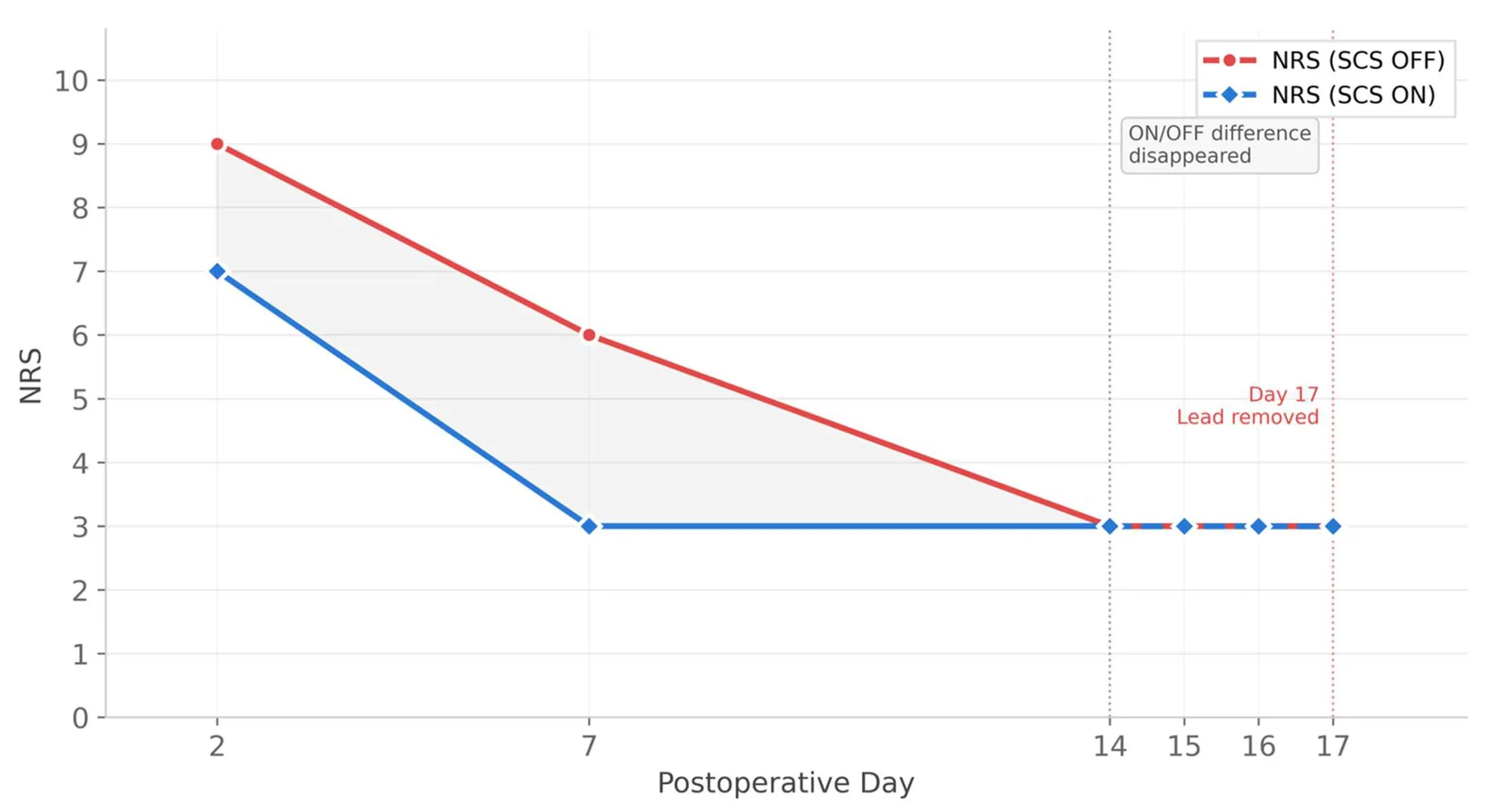
Postoperative course of resting neck pain in Patient 3, assessed by the Numeric Rating Scale (NRS) under SCS ON and OFF conditions. A clear stimulation-dependent reduction in pain was observed in the early recovery period (days 2 and 7). On day 14, the ON/OFF difference disappeared, indicating that the analgesic contribution of SCS had resolved in parallel with natural postoperative recovery. Leads were removed on day 17.

Following C2-7 open-door laminoplasty under general anesthesia, two 16-contact percutaneous SCS leads were placed at the C2-5 levels during the same anesthetic (Figure 1). Postoperative resting pain was assessed under SCS ON and OFF conditions (Table 1, Figure 2).

### 2.4. Postoperative Assessment Protocol

In all patients, ON/OFF assessment was repeated during postoperative programming, confirming stimulation-specific pain relief. Assessments were unblinded: the order of testing was OFF–ON–OFF–ON, performed twice at each timepoint, with stimulation set to ON as the default state between assessments. Pain ratings were obtained approximately 10–15 min after each switch between stimulation conditions, allowing sufficient time for the patient to perceive the change in stimulation. Pain ratings were obtained directly from each patient by the treating physician (also the investigator). Stimulation settings were individually adjusted. The evaluated parameters were pain on a numerical rating scale (NRS) (preoperatively; postoperative day 2 SCS OFF/ON; and at final evaluation 6 months after SCS initiation or at lead removal), time to mobilization, lead outcome, and adverse events.

## 3. Results

The clinical course of all five patients is summarized in Table 1. All patients were mobilized on postoperative day 1. The mean NRS decreased from 9.6 preoperatively to 8.6 with SCS OFF and 6.6 with SCS ON on postoperative day 2 and to 3.6 at final evaluation. No procedure-related adverse events, including infectious complications, occurred.

In patients 1, 2, and 5, continued stimulation benefit was observed, and an IPG was implanted for permanent use. Follow-up durations for these three patients were 4 years 7 months, 1 year 10 months, and 1 year 6 months, respectively; in all three, stimulation settings required only minor adjustments, and therapeutic benefit remained stable throughout follow-up. In patients 3 and 4, the therapeutic objective was achieved during the acute postoperative period, and the leads were electively removed; no recurrence of pain was observed after removal.

In the representative case (Patient 3), on day 2, NRS was 9 (OFF) and 7 (ON); on day 7, NRS was 6 (OFF) and 3 (ON), confirming stimulation-dependent pain relief (Figure 2). On day 14, NRS was 3 under both ON and OFF conditions; leads were removed on day 17. The mJOA score at discharge was 16. The patient returned to work approximately six weeks postoperatively, and neck pain was NRS 2 at 12-month follow-up.

In this small series, although the individual contribution of SCS to early mobilization cannot be quantified, introducing neuromodulation at the time of surgery may help reduce the barrier of acute postoperative pain to rehabilitation in carefully selected patients.

## 4. Discussion

### 4.1. Clinical Necessity: The Significance of Timing

A prerequisite for this approach is that the patient already has an established SCS indication at the time of spine surgery, with chronic and inadequately controlled pain. In these patients, introducing SCS at the time of spinal surgery, rather than waiting until the postoperative period, carries clear clinical rationale. In OPLL, severe preoperative neck pain may persist postoperatively [3,4,5]. In multilevel degenerative disease, the extent of the surgical wound and complexity of neural involvement complicate postoperative pain management [6]. In PD with concomitant spinal disease, complication rates after decompression are higher [7,10], making it important to maximize the therapeutic value of each surgical opportunity. Simultaneous SCS introduction allows neuromodulation to be delivered precisely when it is most needed.

In this context, “simultaneous” refers specifically to SCS lead placement performed under the same anesthetic as the spine surgery, with the intent of avoiding an additional invasive procedure and its associated procedural risks and reducing the overall burden on the patient. It does not imply that SCS is offered pre-emptively to patients without an established indication; rather, it describes the timing of an already-indicated intervention.

### 4.2. Lower Overall Invasiveness

Completing spinal surgery and SCS lead placement in a single operation and under a single anesthetic reduces the overall surgical burden on the patient. The need for a separate percutaneous SCS procedure is eliminated, avoiding duplication of anesthetic risk.

### 4.3. Lead Placement Accuracy and Stability

Conventional percutaneous SCS placement is performed from the body surface without direct visualization of the epidural space. When performed simultaneously with spine surgery, direct visualization of the epidural entry site may facilitate lead introduction, while fluoroscopy is required to guide advancement and confirm final lead position. This combined approach may be advantageous in anatomically altered epidural spaces after decompression. In the cervical spine in particular, advancing the lead tip to the C2 level may help stabilize the lead even in an epidural space altered by decompression [11]. Sixteen-contact leads were selected to enable broader spinal coverage.

### 4.4. The Significance of Reversibility

The reversibility of the percutaneous lead is fundamental to this approach [2]. Because it is reversible, SCS can be introduced with low invasiveness when clinically necessary, and management can then be adjusted according to the subsequent clinical course. In these five patients, the duration of required stimulation differed among individuals. In two patients, the therapeutic objective was achieved during the acute postoperative period, and the lead was removed without recurrence [12]. In three patients, the benefit of stimulation persisted, and treatment transitioned to permanent use. Neither the timing of SCS initiation nor the required duration of therapy is uniform; reversible leads can accommodate this variability. Early introduction of SCS may also carry broader implications for outcomes, including early mobilization, return to social activity, and patient satisfaction [13].

An important ethical prerequisite for this approach is that patients already have an established indication for SCS at the time of spine surgery, with chronic and inadequately controlled pain that has not responded sufficiently to conservative measures. The technique is not intended as a pre-emptive or prophylactic intervention in patients without a clear SCS indication. Rather, it leverages the existing surgical opportunity to introduce a reversible neuromodulatory therapy when it is most needed, while preserving the option to discontinue stimulation and remove the leads once their role has been fulfilled.

### 4.5. Limitations

This report represents a retrospective observation of five patients at a single institution by a single surgeon, and no generalization is possible. There was no control group, and the individual contribution of SCS to outcomes cannot be quantified.

ON/OFF assessments were unblinded, and expectation and placebo effects cannot be excluded. The observed pain reduction may also reflect the effects of decompressive surgery itself, natural postoperative recovery, and perioperative analgesics, and should not be interpreted as evidence of an SCS-specific effect in isolation. Similarly, given the absence of a comparator group, this study cannot establish that this approach directly contributed to early mobilization. Although no infectious complications occurred in this series, percutaneous lead externalization inherently carries an infection risk, and the small sample size limits the ability to detect uncommon complications. The four underlying conditions included in this cohort are clinically heterogeneous; while this breadth illustrates the range of potential applications, it may also limit the consistency of the results and their interpretation. All patients received conservative treatment with medication alone prior to SCS, without escalation to interventional treatments such as nerve blocks, which represents a further limitation in patient selection. Intraoperative neurophysiological confirmation of lead position was not performed in this series; lead position was instead confirmed by intraoperative fluoroscopy and postoperative CT. Beyond NRS, no other neurological outcome measure common to all five patients was systematically recorded, given the heterogeneity of underlying conditions and the disease-specific nature of standard neurological assessment tools; this precluded a more detailed pre- to postoperative neurological comparison across the cohort.

Long-term outcomes and cost-effectiveness were not assessed. This experience was obtained within the Japanese national health insurance framework, in which this approach falls within the established coverage for SCS; individual evaluation against the relevant reimbursement framework will be required in other healthcare systems.

## 5. Conclusions

Simultaneous percutaneous SCS lead placement during spine surgery may allow neuromodulation to be delivered at the time it is needed, for the duration it is required, and at the relevant spinal level. The approach combines three practical advantages: it avoids a separate surgical procedure, facilitates lead introduction through direct visualization of the epidural entry site, and preserves full flexibility through reversibility. In carefully selected patients with an established SCS indication at the time of spine surgery, this technique warrants further prospective evaluation.

## Figures and Tables

**Table 1:** Summary of patients.

	Patient 1	Patient 2	Patient 3	Patient 4	Patient 5
**Age/Sex**	76/F	85/M	56/M	77/M	78/M
**Condition**	PD + multilevel spinal pain	Multilevel cervical and lumbar degenerative disease	Cervical OPLL	Multilevel cervical and lumbar degenerative disease	PD + lumbar spinal canal stenosis
**Pain character**	Neuropathic	Neuropathic	Neuropathic	Neuropathic	Neuropathic
**Duration (months)**	36	60	3	9	18
**Prior conservative treatment**	Medication only	Medication only	Medication only	Medication only	Medication only
**Spine surgery**	Lumbar decompression	Cervical laminoplasty → lumbar decompression	Cervical laminoplasty	Cervical laminoplasty → lumbar decompression	Lumbar decompression
**Lead (contacts; level)**	8-contact ×2/C2–4, T9–11	16-contact ×2/C6–T3, T8–11	16-contact ×2/C2–5	16-contact ×2/C2–5, T8–11	16-contact ×2/T8–11
**Manufacturer**	Medtronic	Boston Scientific	Boston Scientific	Boston Scientific	Boston Scientific
**Pulse width (μs)**	300	90	450	300	200
**Frequency (Hz)**	4	300	50	50	5
**Preoperative NRS**	10	9	10	9	10
**POD 2: SCS OFF/ON**	8/5	9/7	9/7	8/7	9/7
**Final NRS (timepoint)**	2 (6 months)	4 (6 months)	3 (POD17)	5 (POD21)	4 (6 months)
**Mobilization**	POD1	POD1	POD1	POD1	POD1
**Outcome**	Permanent use; stable at 4y7m follow-up	Permanent use; stable at 1y10m follow-up	Acute-phase only; removed POD17	Acute-phase only; removed POD21	Permanent use; stable at 1y6m follow-up
**Recurrence after removal**	—	—	None	None	—
**Adverse events**	None	None	None	None	None

POD, postoperative day; NRS, numerical rating scale; OPLL, ossification of the posterior longitudinal ligament; PD, Parkinson’s disease. “POD 2: SCS OFF/SCS ON” gives the NRS with stimulation OFF and ON at the same single time point on postoperative day 2. In patients 3 and 4, the ON/OFF difference had disappeared at the time of lead removal. All patients received prophylactic antibiotics and daily wound inspection during lead externalization; no infectious complications occurred. Lead position was confirmed intraoperatively by fluoroscopy and postoperatively by CT in all cases.

## Data Availability

The data presented in this study are available on request from the corresponding author.

## Abbreviations

The following abbreviations are used in this manuscript:

SCS	spinal cord stimulation;
OPLL	ossification of the posterior longitudinal ligament
PD	Parkinson’s disease
NRS	numerical rating scale
IPG,	implantable pulse generator
mJOA	modified Japanese Orthopaedic Association
POD	postoperative day

## References

[1] Jeon Y.H. (2012). Spinal Cord Stimulation in Pain Management: A Review. Korean J. Pain.

[2] Shanthanna H., Eldabe S., Provenzano D.A., Bouche B., Buchser E., Chadwick R., Doshi T.L., Duarte R., Hunt C., Huygen F.J.P.M. (2023). Evidence-based consensus guidelines on patient selection and trial stimulation for spinal cord stimulation therapy for chronic non-cancer pain. Reg. Anesth. Pain Med..

[3] Nagoshi N., Yoshii T., Egawa S., Sakai K., Kusano K., Tsutsui S., Hirai T., Matsukura Y., Wada K., Katsumi K. (2022). Clinical Indicators of Surgical Outcomes After Laminoplasty for Patients With Cervical Ossification of the Posterior Longitudinal Ligament: A Prospective Multicenter Study. Spine.

[4] Koda M., Yoshii T., Egawa S., Sakai K., Kusano K., Nakagawa Y., Hirai T., Wada K., Katsumi K., Kimura A. (2021). Factors Significantly Associated with Postoperative Neck Pain Deterioration after Surgery for Cervical Ossification of the Posterior Longitudinal Ligament: Study of a Cohort Using a Prospective Registry. J. Clin. Med..

[5] Oshima Y., Matsubayashi Y., Taniguchi Y., Hayakawa K., Fukushima M., Oichi T., Oka H., Riew K.D., Tanaka S. (2018). Mental State Can Influence the Degree of Postoperative Axial Neck Pain Following Cervical Laminoplasty. Glob. Spine J..

[6] Wang S.-J., Jiang S.-D., Jiang L.-S., Dai L.-Y. (2010). Axial pain after posterior cervical spine surgery: A systematic review. Eur. Spine J..

[7] Westermann L., Eysel P., Hantscher J., Baschera D., Simons M., Herren C., Zarghooni K., Siewe J. (2017). The Influence of Parkinson Disease on Lumbar Decompression Surgery: A Retrospective Case Control Study. World Neurosurg..

[8] Daniels A.H., McDonald C.L., Basques B.A., Hershman S.H. (2022). Perioperative Management of Spinal Cord Stimulators and Intrathecal Pain Pumps. J. Am. Acad. Orthop. Surg..

[9] Rigoard P., Ounajim A., Goudman L., Wood C., Roulaud M., Page P., Lorgeoux B., Baron S., Nivole K., Many M. (2022). Combining Awake Anesthesia with Minimal Invasive Surgery Optimizes Intraoperative Surgical Spinal Cord Stimulation Lead Placement. J. Clin. Med..

[10] Sarica C., Zemmar A., Yousefi O., Yang A.C., Uzuner A., Sheng Z., Santyr B., Samuel N., Colditz M., Vetkas A. (2023). Spinal Cord Stimulation for Parkinson’s Disease: A Systematic Review and Meta-Analysis of Pain and Motor Outcomes. Ster. Funct. Neurosurg..

[11] Deer T.R., Mekhail N., Provenzano D., Pope J., Krames E., Leong M., Levy R.M., Abejon D., Buchser E., Burton A. (2014). The Appropriate Use of Neurostimulation of the Spinal Cord and Peripheral Nervous System for the Treatment of Chronic Pain and Ischemic Diseases: The Neuromodulation Appropriateness Consensus Committee. Neuromodulation Technol. Neural Interface.

[12] Lee S.J., Yoo Y.M., A You J., Shin S.W., Kim T.K., Abdi S., Kim K.H. (2019). Successful removal of permanent spinal cord stimulators in patients with complex regional pain syndrome after complete relief of pain. Korean J. Pain.

[13] Kobayashi R., Taketomi A., Hara E., Mera H., Oe K. (2024). Temporary Spinal Cord Stimulation for Herpes Zoster With Myelitis: A Case Series. Cureus.

